# Medical versus neurosurgical treatment in ICH patients: a single center experience

**DOI:** 10.1007/s10072-023-07015-0

**Published:** 2023-08-14

**Authors:** P. Pierini, Agnese Novelli, F. Bossi, R. Corinaldesi, M. Paciaroni, M. G. Mosconi, A. Alberti, M. Venti, I. Leone de Magistris, V. Caso

**Affiliations:** 1 Department of Emergency Medicine, Città Di Castello Hospital, Città Di Castello, Italy; 2 Internal, Vascular and Emergency Medicine-Stroke Unit, Santa Maria della Misericordia University of Perugia, 06139 Perugia, Italy; 3 Neurosurgery Department, Santa Maria Della Misericordia Hospital, Perugia, Italy; 4 Stroke Unit, Santa Maria Della Misericordia, University of Perugia, Perugia, Italy

**Keywords:** Neurosurgery, Surgery, ICH, Haemorrhage, Therapy

## Abstract

**Background and aims:**

The effect of surgical treatment for spontaneous intracerebral hemorrhage (ICH) remains uncertain. We conducted an observational retrospective cohort study on supra-centimeter spontaneous ICH treated with either neurosurgical or conservative management. The baseline demographics and risk factors were correlated with in-hospital mortality and 3 and 6-month survival rates stratified by management.

**Methods:**

We included all patients with evidence of spontaneous ICH > 1 cm detected by CT and admitted between august 2020 and march 2021 to the “SMM” Hospital in Perugia.

**Results:**

Onehundredandtwentytwo patients were included in the study, and 45% (n.55) were surgically treated. The mean age was 71.9 ± 15.3, and 61% (n.75) were males. Intra-hospital mortality ended up being 31% (n.38), 3 months-survival was 63% (n.77) and 6 months-survival was 60% (n.73).

From the multivariate analysis of the surgical patients versus medical patient, we observed that the surgical patients were younger (67.5 ± 14.9 vs 75.5 ± 14.7 y; OR 0.87; Cl 95% 0.85–0.94; p 0.001), with greater ICH volume at the onset (61 ± 39.4 cc vs 51 ± 64 cc; OR 1.03; Cl 95% 1.005–1.07; p 0.05), more midline shift (7.61 ± 5.54 mm vs 4.09 ± 5.88 mm; OR 1.37; Cl 95% 1.045–1.79; p 0.023), and a higher ICH score (3 vs 2 mean ICH score; OR 21.12; Cl 95% 2.6–170.6; p 0.004). Intra-hospital mortality in the surgical group and in the conservative treatment group was respectively 33% vs 30%, 3 month-survival was 64% vs 63% and 6 month- survival were 60% in both groups.

**Conclusions:**

Our patient cohort shows no overall benefit from surgery over conservative treatment, but surgical patients were younger and had larger ICH volume.

## Introduction

The American Stroke Association defines intracerebral hemorrhage (ICH) as “the rapid development of neurological signs and symptoms attributable to the accumulation of blood within the brain parenchyma or ventricles not caused by trauma”; accounting for 10- 20% of all strokes and responsible for disproportionately high morbidity and mortality rates worldwide [1–3]. Although 30-day case fatality significantly decreased over the last decades, additional improvements in the acute management of ICH are needed since very early case-fatality rates (within 48 h) did not improve [4] compared to ischemic stroke. Moreover, the 30-day ICH outcome improvement has been recorded only in high-income countries [1]*.* Current international guidelines on the management of ICH have recommended that ICH be treated as a medical emergency by acting on the neurological and non-neurological complications of ICH in order to reduce mortality. However, unlike with ischemic strokes, there is no specific treatment for ICH.

The benefit of surgical treatment for ICH remains uncertain. STICH I [5] showed no overall benefit from early surgery compared to initial conservative treatment. In contrast, STICH II [6] reported that early surgery does not increase the rate of death or disability at 6 months and might also have a small but clinically relevant survival advantage for patients with spontaneous superficial ICH without intraventricular hemorrhage. In both studies, when it comes to neurologic deterioration, there was a high crossover rate between the medical management and the surgical group. These crossovers may have led to a dilution of any possible clinical benefit deriving from surgical intervention. The most recent systematic review and meta-analysis of 21 randomized clinical trials, involving the surgical treatment of supratentorial ICH, showed that the likelihood of a good functional outcome was 40% higher in patients undergoing any surgical intervention than in those who had not received it.

Surgical treatment of supratentorial spontaneous ICH may be beneficial, in particular when using minimally invasive procedures and when performed soon after symptom onset [7, 8].

Still, the indications for surgery are not uniformly adopted by stroke centers, leading to case-by-case decisions based on the treating physician's discretion [9, 10]*.* However, surgical patients have been reported to show a better survival rate, though not a better functional outcome [11]. Moreover, surgical patients tend to be followed up less frequently and therefore long-term data is missing.

Taking these premises into account, we conducted an observational retrospective cohort study in our own center, focusing on supracentimetric spontaneous ICH treated either with neurosurgical treatment or with conservative management comparing the outcome of the two groups.

## Methods

Between August 2020 and March 2021, supracentimetric spontaneous ICH, treated with neurosurgical or conservative management, of patients admitted to the Intensive Care Unit, Stroke Unit, Internal and Vascular Medicine, Neurosurgery, Neurology and Geriatrics departments at the Santa Maria della Misericordia Hospital in Perugia (Italy), were prospectively collected and retrospectively analysed.

The recruitment included all patients with evidence of supracentimetric ICH (> 1 cm) found through head computed tomography, detected by the IMPAX radiology software in use at the Perugia hospital. Recruitment included all patients with the diagnostic code of 431 ICD-9-CM, either at admission or discharge whereas surgically treated patients were extracted from ORMAWEB (surgical computer archive).

Exclusion criteria were: primary subdural/epidural hematoma, traumatic ICH, hemorrhage due to cancer (nonvascular origin), primary subarachnoid hemorrhage (with or without ICH), haemorrhagic transformation of cerebral infarction (with or without thrombolysis), ICH with vascular malformation and Cerebral Venous Thrombosis. The Perugia Stroke Team follows a stepwise routine protocol of neuroimaging for ICH patients [12].

Data regarding ICH risk factors were collected for each patient, including age, sex, location, volume measured by the ABC/2 method [13] (to estimate ICH volume, A = maximum length in cm, B = width perpendicular to A on the same head CT slice, and C = the number of slices multiplied by the slice thickness), blood pressure, anticoagulation reversal therapy, GCS, and ICH score. History of hypertension (hypertension was defined BP > 140/90 mmHg twice before the event or patient being currently on anti-hypertensive treatment), amyloid angiopathy (the Modified Boston criteria were used to define amyloid angiopathy), primary brain tumors, myocardial infarction, previous ICH or ischemic stroke, diabetes mellitus (pre-prandial glycaemia ≥ 126 mg/dL on at least 2 examinations, post-prandial glycaemia ≥ 200 mg/dL, HbA1c ≥ 6.5% or patient currently following hypoglycemic treatment), obesity (body mass index ≥ 30 kg/m2), hyperlipidemia (total cholesterol ≥ 200 mg/dL or triglyceride ≥ 140 mg/dL or already under lipid lowering therapy), ongoing smoking, alcohol abuse (≥ 300 g per week), drugs, migraine, migraine with aura, history of symptomatic peripheral arterial disease (intermittent claudication of presumed atherosclerotic origin; or ankle/arm systolic blood pressure ratio < 0.85 in either leg at rest; or history of intermittent claudication with previous leg amputation, reconstructive surgery, or angioplasty), atrial fibrillation (AF) and/or systemic diseases (thrombocytopenia, bleeding disorders, kidney failure, liver disease).

Data on oral anticoagulants (OAT), direct oral anticoagulants (DOAC), heparin, and antiplatelet agents were collected.

At admission, the severity of ICH was measured by the ICH-score [14] and surgical techniques were recorded.

Data regarding intra-hospital mortality rates and length of hospitalization was recorded for each patient. Follow-up visits and outcome adjudication were performed, not in a blinded fashion. Follow up was performed when possible, in our outpatient clinic or by phone through a structure interview [15].

Differences between the characteristics of ICH classified in surgical or conservative management and the characteristics of deceased and non-deceased patients were tested using the chi-Square test for categorical variables. Analyses resulting in values of *p* < 0.05 were considered significant.

Different logistic regression models (multivariable analyses) were used as a second step to verify the predictive factors associated with the neurosurgical intervention and mortality.

The following variables were of interest:

A) for the first model (predictive factors associated with surgery), in addition to clinical characteristics of patients (age, sex, and severity), variables that were included consider the characteristics of the ICH (anterior versus posterior, lobar or deep seated, lesion size, midline shift, presence of edema, hydrocephalus, intraventricular invasion). The ICH score was also included in the model.
B) in the second model (predictive factors associated with mortality) the clinical characteristics of the patients (like sex, age, diabetes, AF, previous ICH, MI or stroke), the characteristics of the bleeding, the ICH score, and surgery were included.

The Odds Ratio (OR) with 95% confidence intervals was used for the obtained results.

## Results

A total of 163 surgical patients were identified based on the 431 ICD-9 code, 41 patients were excluded because they had traumatic ICH, MAV or aneurysm.

A total of 122 patients were included in the study, and 45% were surgically treated. The type of surgical approach is listed in Table 1.

**Table 1:** Type of surgical treatment

	Total	Surgical
Surgery	55 (45%)	
Craniotomy		40 (73%)
EVD		12 (22%)
Craniotomy + EVD		3 (5%)

Concerning the ICH locations, most recorded cases were lobar and a smaller number was found to be located in the brainstem. (Table 2).

**Table 2:** ICH location and main presentations at onset

Location	Total	Surgical	Conservative	*p*-value
Right	68 (56%)	34 (62%)	34 (51%)	0.22
Left	52 (43%)	20 (36%)	32 (48%)	0.20
Central	2 (2%)	1 (2%)	1 (1%)	0.88
Lobar	66 (54%)	31 (56%)	35 (52%)	0.64
Deep	38 (31%)	13 (24%)	25 (37%)	0.10
Cerebellar	15 (12%)	10 (18%)	5 (7%)	0.07
Brainstem	3 (2%)	1 (2%)	2 (3%)	0.67
Supratentorial	104 (85%)	44 (80%)	60 (90%)	0.13
Infratentorial	18 (15%)	11 (20%)	7 (10%)	0.13
Presentation at onset	Total	Surgical	Conservative	*p*-value
IVH	50 (41%)	27 (49%)	23 (34%)	0.09
Hydrocephalus	37 (30%)	22 (40%)	15 (22%)	0.035
Herniation	13 (11%)	8 (15%)	5 (7%)	0.2
Mean volume (cc)	55.47	61 ± 39.4	51 ± 64	0.3
Mean ICH score	2.2	3	1.8	0.04
ICH score 0	21 (17%)	2 (4%)	19 (28%)	0.0003
ICH score 1	21 (17%)	3 (5%)	18 (27%)	0.0018
ICH score 2	25 (20%)	18 (33%)	8 (12%)	0.005
ICH score 3	20 (16%)	13 (24%)	7 (10%)	0.05
ICH score 4	12 (10%)	6 (11%)	7 (10%)	0.93
ICH score 5	9 (7%)	3 (5%)	6 (9%)	0.46
ICH score 6	2 (2%)	1 (2%)	1 (1%)	0.88
Midline shift (mm)	5.58 ± 5.98	7.61 ± 5.54	4.09 ± 5.88	0.001
Mean systolic BP (mmHg)	159 ± 33	171 ± 39	153 ± 29	0.019
Mean diastolic BP (mmHg)	89 ± 16	93 ± 20	87 ± 14	0.099
mRS	1.3	0.7	1.7	0.04
Reversal therapy	14 (11%)	7 (13%)	7 (10%)	0.69

The ratio of male and female patients was the same in both groups, the surgical patients were younger, but no statistically significant difference was found between the two groups when it comes to risk factors and antithrombotic therapy. (Table 3).

**Table 3:** Characteristics and risk factors

Characteristics	Total	Surgical	Conservative	*p*-value
	122	55	67	
Male	75 (61%)	34 (62%)	41 (61%)	0.94
Female	47 (39%)	21 (38%)	26 (39%)	0.94
Mean age (y)	71.9 ± 15.3	67.5 ± 14.9	75.5 ± 14.7	0.004
Risk Factors	Total	Surgical	Conservative	*p*-value
Anticoagulant	32 (26%)	14 (25%)	18 (27%)	0.86
Antiplatelet	34 (28%)	15 (27%)	19 (28%)	0.89
Hypertension	89 (73%)	39 (71%)	50 (75%)	0.64
Amyloid angiopathy	4 (3%)	1 (2%)	3 (4%)	0.41
Alcohol	3 (2%)	1 (2%)	2 (3%)	0.67
Venous thrombosis	1 (1%)	0	1 (1%)	
Diabetes	27 (22%)	9 (16%)	18 (27%)	0.16
Previous MI	12 (10%)	6 (11%)	6 (9%)	0.71
AF	39 (32%)	14 (25%)	26 (39%)	0.11
Previous stroke	25 (20%)	8 (15%)	17 (25%)	0.14
Previous ICH	16 (13%)	8 (15%)	8 (12%)	0.67
Cognitive impairment	31 (25%)	10 (18%)	21 (31%)	0.09
Covid	1 (1%)	0	1 (1%)	
Liver disease	5 (4%)	4 (7%)	1 (1%)	0.10
Obesity	11 (9%)	4 (7%)	7 (10%)	0.54
Smoke	8 (7%)	4 (7%)	4 (4%)	0.77
Chronic Kidney Disease	11 (9%)	4 (7%)	7 (10%)	0.54
Tumors	10 (8%)	6 (11%)	4 (6%)	0.32
Hematological tumors	2 (2%)	2 (4%)	0	
Antithrombotic Therapy	Total	Surgical	conservative	*p*-value
Antiplatelet	31 (25%)	15 (27%)	16 (24%)	0.66
VKA	10 (8%)	2 (4%)	4 (6%)	0.55
Heparin products	4 (3%)	2 (4%)	2 (3%)	0.84
DOAC	16 (13%)	6 (11%)	10 (15%)	0.51
Association	3 (2%)	0	3 (4%)	

Within the intensive care unit, the difference between the number of patients treated surgically and the number of patients treated with a conservative approach was statistically significant, as the former group was considerably higher. Patients who received conservative treatment had been admitted to the stroke unit and in neurosurgery in equal percentages, but the hospital stay lasted longer in the neurosurgery department because of the more frequent infections. In terms of survival, there is no difference between the two groups (Table 4).

**Table 4:** Hospitalization departments, non-neurological complications and outcomes

	Total	Surgical	Conservative	*p*-value
Intensive Care Unit	45 (37%)	42 (76%)	3 (4%)	< 0.0001
Stroke unit	27 (22%)	7 (13%)	20 (30%)	0.023
NCH	49 (40%)	27 (49%)	22 (33%)	0.068
Other	24 (20%)	3 (5%)	21 (31%)	0.003
Mean Hospital stay (days)	16 ± 15	21 ± 18	12 ± 9,5	< 0.00001
Infectious complications	64 (52%)	37 (67%)	27 (40%)	0.002
Thrombotic complications	35 (29%)	27 (49%)	8 (12%)	< 0.00001
Mechanical ventilation	37 (30%)	34 (62%)	3 (4%)	< 0.00001
Tracheostomy	20 (16%)	20 (36%)	0	
PEG	5 (4%)	5 (9%)	0	
Seizures	23 (19%)	12 (22%)	11 (16%)	0.44
Outcomes	Total	Surgical	Conservative	*p*-value
In-hospital death	38 (31%)	18 (33%)	20 (30%)	0.73
3 months-survival	77 (63%)	35 (64%)	42 (63%)	0.91
6 months-survival	73 (60%)	33 (60%)	40 (60%)	0.97
Dead at follow-up	59 (48%)	30 (55%)	43 (64%)	0.28
Survival mean (months)	3.35	5.46	1.17	< 0.00001

In the first model, the predictive factors associated with surgical management were the age, the volume at the onset, the midline shift seen with neuroimaging, the ICH score and the systolic BP when they arrived at the Emergency department.

In the second model, the predictive factors associated with mortality at six months were volume and history of previous ICH (Table 5).

**Table 5:** Predictive factors associated with surgical management and mortality

I model	OR (Odds Ratio)	95% CI	*p*-value
Age	0.87	0.80–0.94	0.001
Lobar	12.53	0.53–292.5	0.11
Volume	1.034	1.005–1.07	0.05
Shift	1.37	1.045–1.79	0.023
IVH	0.19	0.16–2.25	0.18
Hydrocephalus	1.6	0.2–12.84	0.65
Herniation	0.58	0.55–6.20	0.65
GCS	1.48	0.00–2.20	0.51
ICH score	21.12	2.6–170.6	0.004
Location	0.73	0.24–2.20	0.57
Deep	2.51	0.16–39.32	0.51
Sex Male	0.37	0.082–1.69	0.2
Systolic BP	1.052	1.022–1.083	0.001
II model	OR (Odds Ratio)	95% CI	*p*-value
Age	1.05	0.97–1.15	0.18
Lobar	0.76	0.11–50.77	0.9
Volume	1.02	1.00–1.05	0.032
ICH score	1.6	0.43–5.85	0.47
Deep	2.58	0.55–120.15	0.62
Sex Male	1.19	0.21–6.55	0.83
Systolic BP	1.00	0.98–1.03	0.56
Surgery	1.35	0.20–9.17	0.75
Diabetes	0.67	0.6–7.60	0.75
Previous MI	1.03	0.37–29.03	0.98
Previous stroke	1.2	0.94–15.43	0.88
Previous ICH	9.19	1.00–84.39	0.05
AF	3.46	0.43–27.46	0.23

## Discussion

Our patient cohort with spontaneous supracentimetric ICH showed no statistically significant improvement in the surgery outcome when compared to conservative treatment.

The results are in line with those published by STICH II, which reported that early surgery does not increase the rate of death or disability after six months.

In our study a high mortality rate of 31% was recorded, which was in line with other studies [16, 17] where in-hospital and 1-year mortality were shown to be high (32.4% and 45.4%, respectively). The authors of the papers reported that the death rate from ICH has slightly improved over the past 10 years, in opposition to the marked improvement in the prognosis for ischemic stroke. Moreover, in this study, both in-hospital and 1-year mortality decreased by 10.4% (from 37.5% to 27.1%, *P* < 0.001) and 7.6% (from 50.0% to 42.4%, *P* < 0.001) respectively; This was probably due to better care during acute hospitalization, leading to a subsequent reduction in mortality [16, 17] as both US and Canada ICH patients tend to be more often managed in Neuro-intensive care Units.

We did not observe this reduction in intrahospital mortality compared to our data in 2013 [18], where 62.3% of patients had poor outcomes, including 27.6% deaths after 3 months. In a former analysis conducted by our Center on ICH patients, the aforementioned risk factors for ICH, such as age and high NIHSS at admission, were also significantly associated with intra-hospital mortality [19], which is in line with our current data.

Regarding hospitalization, our mean hospital stay was longer in the neurosurgical group (21 ± 18 days vs 12 ± 9.5 days) with statistically higher complication rates for infections and thrombosis. The European center of infectious diseases (ECDC) reported Italy among the countries in Europe with the highest rate of infections; about 6% of hospitalized patients contract hospital-acquired infections. This higher rate of infections reported in Italian structures may contribute to the risk of offsetting the benefits related to surgical procedures.

Unfortunately, only 22% of ICH patients was admitted in the stroke unit and approximately 33% of patients treated conservatively were admitted to the neurosurgery department, which is not specialized in stroke patients. Stroke units offer a specialized, multidisciplinary approach that has been proven highly beneficial for patients with ICH, providing dedicated care, close monitoring, and interventions tailored to the specific needs of stroke patients, including those with ICH. Studies have consistently shown that care in a stroke unit significantly improves survival and reduces the risk of disability in patients with stroke, including those with ICH. The presence of stroke experts allows for immediate and appropriate management of complications such as increased intracranial pressure or neurological deterioration. Additionally, early rehabilitation by physiotherapists and logopedics facilitates recovery and improves patient short and longterm functional outcomes [20]*.* Therefore, 60% of our patients did not receive the most beneficial treatment [21]*.*

At the follow-up, the mean survival time was longer in the surgically treated patients. However, the longer hospitalization in the surgical group led to a higher incidence of infectious and thrombotic complications, and the need for invasive devices such as PEG and tracheostomy.

Surgical treatment was preferred in the case of younger patients, patients presenting midline shift, patients with higher ICH volume and patients with a higher ICH score at admission. There is a global tendency to overtreat younger patients even when there is no survival prognosis.

Optimal hematoma volume cutoffs for predicting poor outcome in deep ICH vary according to the specific deep brain nucleus involved [22], while older patients tend to not be treated, leading to a later secondary damage. However, ICH are reported to have a greater potential for recovery compared to ischemic stroke after the acute phase when patients recover from primary and secondary damage [23].

Our study has the following strengths:

A) Patients were admitted consecutively to a high-volume Hub center providing insights into the clinical practice related to the acute phase and associated complications.
B) Patients were followed up for a longer time period (> 3 months), providing information on midterm complications and management.

Whereas the limitations of the study are the following:

A) The study was not randomized but based on the discretion of the single physician.
B) We did not calculate the sample size to accurately show the efficacy of surgical treatment.

## Conclusion

Our patient cohort with spontaneous supracentimetric ICH showed no overall benefit from surgery compared to conservative treatment. However, we need to consider that surgical patients tended to be younger, presenting more severe clinical conditions, larger ICH volume and a more pronounced midline shift. Further evidence is needed for a more targeted selection of patients aimed at the standardization of surgical treatments and these patients need to be managed in a dedicated stroke unit.

## Data Availability

The data that support the findings of this study are available from the corresponding author (A. N.) upon reasonable request.
